# Optimization of tourism routes in Lushunkou District based on ArcGIS

**DOI:** 10.1371/journal.pone.0264526

**Published:** 2022-03-11

**Authors:** Qian Pei, Li Wang, Peng Du, Zhaolan Wang

**Affiliations:** School of Geography, Liaoning Normal University, Dalian, Liaoning, China; National Taiwan University of Science and Technology, TAIWAN

## Abstract

With the advancements and developments in China’s tourism industry, various autonomous forms of tourism have been gaining prominence. As such, to facilitate tourists and provide them with maximum experience while economizing on time and cost is essential. One approach toward achieving this is the optimization of tourism routes. However, so far the studies on this approach have focused primarily on inland tourist sites and have lacked a geographic perspective. Therefore, this study undertook the tourism resource data of Lushunkou District of 2020, used the ArcGIS accessibility evaluation model to analyze tourism resources, and finally used the Vehicle Routing Problem of network analysis technology to optimize the tourism route of Lushunkou District and obtain the general overall intellectual framework and technical methods for tourism route optimization. The results showed that the ArcGIS accessibility evaluation model could be used to integrate resources in the tourism area before using the Vehicle Routing Problem to optimize the analysis of tourism routes, thereby enabling the separation of different types of tourism. These divisions were based on the Vehicle Routing Problem to optimize routes for one-day and two-day tours. A new method and model for optimization for tourism routes was constructed to provide a basis and reference for the optimization of tourism routes in similar cities. The observations and results of the present study can facilitate the government in developing the tourism industry and maximizing the benefits obtained from them. Further, travel agencies and tourists will have the provision of designing optimum tourism routes.

## Introduction

The tourism industry makes a vital contribution toward the national economy. According to one report, prior to the Covid-19 pandemic, tourism accounted for 1 in 4 of all new jobs created across the world, 10.6% of all global jobs (334 million), and 10.4% of global GDP (9.2 trillion). In 2020, the tourism sector suffered a loss of almost 4.5 trillion, to reach 4.7 trillion [1]. When the epidemic in China is alleviated, all regions should take full advantage of their tourism resources. For tourism enterprises, it is important to provide reasonable tourist routes and different types of scenic spots to attract more tourists and enhance the charm and popularity of scenic spots [2]. The development of tourism should be resumed to avoid greater losses in this sector. By resuming the development of the tourism industry through optimization of tourism routes, not only can we avoid greater losses, but also we can promote compliance with the social distancing measures imposed for Covid-19 control [3, 4].

With the booming tourism industry and the improvement of living standards, tourists pay more and more attention to the tourism experience, with a focus on tour continuity and the environment of the tourist destination. To develop tourism, we should first focus on the effectiveness of tourism experience, analyze the influencing factors (trip factor and time factor) that tourists may be subjected to in the destination [5]. In particular, there is a need to plan tour routes that maximize the tourism experience, while considering tourists’ preference of attraction, time, and cost budgets [6]. Therefore, this article focuses on the sustainability of the tourist experience, that is, the integration of all the tourism resources in a region, and the creation of an optimal recommended route.

The planning of coastal tourism routes can be divided into three parts from the regional scope, which includes planning of tourist routes between cities, the planning of tourist routes in various scenic spots within the city, and scenic spots in coastal travel-route planning [7]. The service objects of tourism route optimization include tourist (single or group) and tourism planning. In recent years, scholars (both in China and abroad) have extensively researched the optimization of tourist routes, and have proposed many research methods. The research methods involved have made use of various algorithms such as the ant colony algorithm [8, 9], genetic algorithm [9], degenerate algorithm [10], probability prediction algorithm [11], data mining [12], backtracking algorithm [3] and so on. Such as optimization of tourist routes based on tourists, Wei Sun used the ant colony algorithm and the cluster analysis to build a model with the shortest time, lowest cost, and best travel experience to study the national 5A-level tourist routes [13]. Wolfgang, W. based on the Dijkstra algorithm, a new method was developed for designing a route composed of users’ preferred scenic spots, according to points of interest [14]. Optimization of tourism routes based on tourism planning, Yan Han proposed an optimal route recommendation mechanism for the prediction of the next tourist attraction and optimal route recommendation to the predicted tourist attraction [2]. Based on GIS network analysis, Gill, N. combined with time and length impedance to estimate the traveling time and distance by useing the shortest path analysis principle, determined the optimal route for the tourist origin to destination places including visiting time at each tourist destination and enhanced potential tourism, thereby increasing the satisfaction of tourists [15]. Investigators from Jeju National University Target Engineering applied a Markov chain model to predict the popularity of different places on the short-and long-term bases [16]. The popularity index alongside user constraints was provided to find optimal routes. These route optimizations were mainly obtained by mining a large amount of route-related data to determine frequently used roads and travel history, which were used to predict new routes. Few researchers have conducted an integrated analysis of tourism resources before conducting such route-optimization studies. In this study, to promote their integrated development, tourism resources are first categorized based on a grid accessibility analysis. Tourism resources that are closer to each other are then integrated, and lower-level tourism resources are also integrated to promote the development of tourism resources that are low in popularity and easily overlooked.

For the regional integration of tourism resources, first of all, we need to understand the types of tourism resources in different areas, such as coastal beach tours, religious and cultural tours, patriotic tours, leisure tours, food shopping tours, etc. These different resources have varying travel categories, such as tour appreciation, knowledge and understanding, personal experience, and recreational vacations. Each region should take full advantage of its own tourism resources, enrich its own tourism categories, and provide a basis for the integration of tourism resources. Tourism resources should be integrated into a large tourist area based on travel time within a certain threshold range and the degree of traffic convenience. To maximize the development of tourism resources, high-level tourism resources in a tourist area should be promoted to lead the development of low-level tourism resources in the same area. Scholars have adopted a method of investigation and analysis for the integrated development of tourism resources, and this article uses GIS accessibility analysis, based on the road network, to calculate the minimum cumulative cost of a certain grid distance from the target grid, to more accurately integrate tourism resources.

This article taking the integrated tourism resources as the destination, based on the cost distance, use Vehicle Routing Problem (VRP) in ArcGIS, set the three necessary attributes of stations, stops, and routes to optimize and analyze travel routes. VRP was first proposed by Dantzig and Ramser [17] in 1959, and since then many research results have been produced on this classical optimization problem [18]. Its application areas are Cold Chain Logistics, healthcare and tourism and so on [19].

In terms of applied research on tourism route optimization, studies have mostly been conducted on a provincial scale; optimization of the tourism spatial structure has been conducted in China’s five northwestern provinces, namely the Ningxia Hui Autonomous Region, Gansu Province [20], Qinghai Province, Xinjiang Uygur Autonomous Region, and Shaanxi Province [21]. Inter-provincial tourism circles were constructed to create an inter-regional tourism network model. Research based on the municipal level has focused on Benxi City [22], Chuzhou City [23], New Delhi City [15], Lokoja [24] and others. These locations have rich tourism resources that must be planned and optimized. The main goal is to develop tourism as a pillar industry, and promote the development of surrounding industries with tourism industry at the center. On the regional scale, scenic spots in Songshan Scenic Area [25], Lushan Scenic Area [26], Gulangyu Scenic Area [27], World Expo Garden Scenic Area [28], forest wetland [9], and other sites have been investigated. The design of tourism routes in these scenic areas has focused on self-driving tours, scientific investigation tours, and other activities. In summary, scholars have mainly focused on the design of northwest cultural tour routes, mountainous natural landscape tours, ecological tour route optimization, and micro-route optimization within scenic areas; however, less emphasis has been placed on the design of coastal city tourist routes.

**Fig 1:**
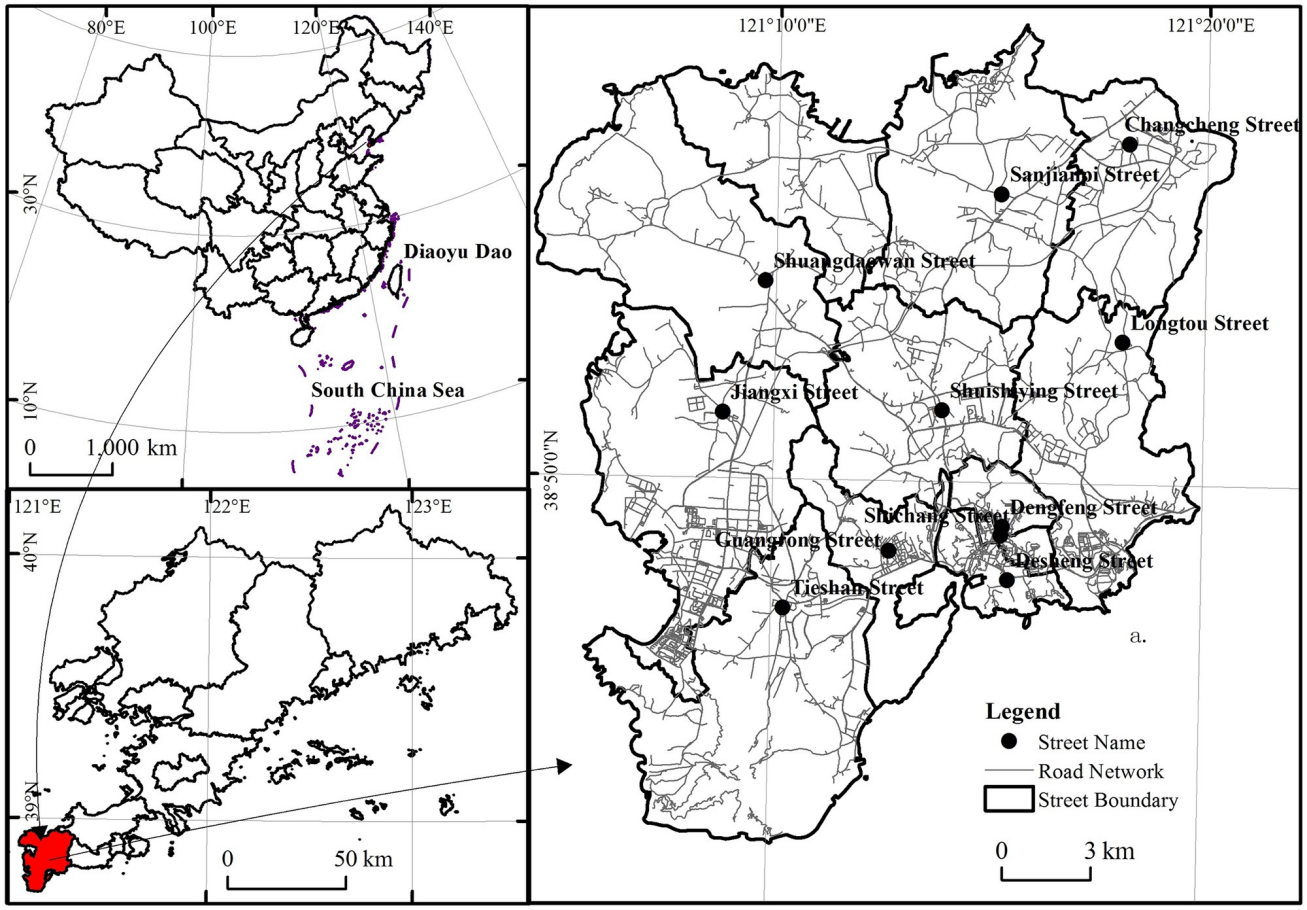
Location and study area: Lushunkou District, in the city of Dalian in Liaoning Province, China.

Coastal regions are a vital tourism attraction, and their superior natural environment and unique location and characteristics have made them a hot-spot for tourism development [29]. In this study, research was conducted at the district/county level to recommend the best travel route to a selected travel location based on the tourism resources of Lushunkou District. The natural ecological environment of Lushunkou District is unique; the district is surrounded by the sea along its eastern, western, and southern sides Fig 1. Lushunkou District comprises an important national strategic point of coastal defense, an important national biodiversity protection area, and a nationally famous modern historical and cultural city. It has not only coastal tourism, but also patriotic tours, leisure tours, scientific and cultural tours and other forms of tourism. It can be used as a typical case area for the development of coastal tourism, to make up for the shortcomings in the optimization of the current coastal tourism route, and to expand the ideas of urban tourism planning in the coastal tourism area.

Given these characteristics of Lushunkou District, the above-mentioned studies and shared bicycles in the tourism resources of Lushunkou District. We used bicycling self-driving tourists as the service object, and constructed the 2020 Lushunkou District Traffic Road Network Data Set. In total, 217 tourism resources were used as research objects, and the accessibility evaluation model in ArcGIS was used to integrate and analyze these resources. Further, the Vehicle Routing Problem (VRP) of network analysis technology was implemented to optimize the tourism routes within the district. By optimizing the tourist route of Lushunkou District, we can obtain the overall thinking framework and technical methods of different forms of tourist route optimization, in order to provide a certain value reference for tourists and the government to optimize tourist routes.

## Data sources and methods

### Data sources and description

This study utilized the traffic road network data of Lushunkou in 2020, which included a national highway, a provincial highway, county roads, township roads, pedestrian roads and other roads. To construct a road network data set in ArcGIS, first, according to the requirements of the “People’s Republic of China Highway Engineering Technical Standards (JT-GB01—2003),” different levels of roads were assigned, as shown in Table 1. Because there is only one railway line to the downtown area of Dalian in Lushunkou District, the tourist route in the district does not involve railways, and only considers the impact of road traffic on the accessibility of scenic spots.

**Table 1:** Composition and speed of the road network in Lushunkou District.

Road class	National highway	Provincial highway	County roads	Township roads	Other roads	Pedestrian roads
Velocity (km/h)	80	60	40	30	20	3
Time (min)	0.75	1	1.5	2	3	10

The tourism resource data of Lushunkou District is sourced from the current tourism resource data from “Bazhuayu” (https://www.bazhuayu.com/) and Baidu POI data. As of 2020, there were a total of 217 tourism resources in this area, including the natural landscape composed of Baiyu Mountain Natural Scenic Area, Snake Island, the “203” scenic area, and the cultural landscape. These cultural sites mainly comprise a Museum group and the site of the Russo-Japanese war; these constitute 12 scenic spots, 98 scenic spots, 64 cultural relics protection units, 26 museums, 6 hot spring sites, 25 coastal tourism resorts, and 50 cherry picking gardens integrated into a network. Based on the national standard “Classification, Investigation and Evaluation of Tourism Resources (GB / T 18972–2003),” the value of ornamental and recreational use of tourism resources in Lushunkou District; the historic cultural, scientific, and artistic prices; cherished peculiarity; scale, demeanor, and probability integrity; popularity and influence; suitable travel period; environmental protection and environmental safety were used to evaluate tourism resources and provide a numerical basis for the following integrated analysis of tourism resources.

According to the evaluation and assignment of tourism resource monomers, as shown in Table 2. There were 6 tourism resources in the fifth class, 39 tourism resources in the fourth class, 45 tourism resources in the third class, 32 tourism resources in the second class, and 96 tourism resources in the first class. Because the number of sightseeing agriculture and hot spring baths in Lushunkou District was large, the number of tourism monomers in the first class was the largest. Moreover, the historic cultural value, scale abundance, popularity, and suitable period of travel for these tourism resources are low, which also result in a large number of monomers in the first class.

**Table 2:** Tourism resources monomer level division standard.

Classification	Score range
Fifth-class tourism resources	≥90
Fourth-class tourism resources	75–89
Third-class tourism resources	60–74
Second-class tourism resources	45–59
First-class tourism resources	30–44
No rank	≤29

### Methods

#### Accessibility evaluation model

The accessibility evaluation model proposed in this study is a grid-consuming distance accessibility analysis method using GIS, which integrates resources based on accumulated travel costs. The cumulative cost of all paths from a grid to the target grid is calculated based on the road network, and the smallest value is assigned to the target grid, which indicates the time cost to pass through the grid. The formulas are as follows [30]:

$$\begin{array}{c} \text{A}=\{\begin{array}{c} \frac{1}{2}\sum_{i+1}^{n}\left(C_{i}+C_{i+1}\right)\\ \frac{\sqrt{2}}{2}\sum_{i+1}^{n}\left(C_{i}+C_{i+1}\right) \end{array}\}\begin{array}{c} \left(a\right)\\ \left(b\right) \end{array} \end{array}$$ (1)

In the formulas, A refers to the accessibility of tourism resources integration, C_i_ refers to the time consumption value of the i-th grid, and C_i+1_ refers to the consumption value of the i+1-th grid along the direction of motion. If the two grids are adjacent in the horizontal or vertical direction, formula (a) is used for the calculation, otherwise formula (b) is used.

#### Vehicle Routing Problem

In this study, tourism routes were optimized to ensure that tours are not repeated, circuits are avoided, and the shortest time is taken during the tour of the attractions. The process of tourism is similar to the Vehicle Routing Problem in “Network Analysis” in ArcGIS, and the model construction in this study was based on the premise that the attractions, their locations, and road network conditions are known, thereby optimizing the tourism routes. The model was composed of three objects: stations, stops, and paths. The station is the one-point type with a fixed location in this model; the stop is a tourist attraction. Tourists depart from the station, visit the stop, and finally return to the station. The purpose was to ensure the shortest distance and the best route for the tour. The construction principles of the VRP are as follows [31]:

In the model, k is the tourist, i is the scenic spot, d_ij_ is the distance between the scenic spot i and j, and W is the workload of the route from the scenic spot i to j; the purpose of the tourist k is to select the scenic spot i_1_, which is furthest away from the site and the shortest distance between i_1_ and i_2_ is (i_1_,i_2_), thereby satisfying:

$$\begin{array}{c} W_{i_{1}}+W_{i_{2}}\leq W \end{array}$$ (2)

There will be any number of branch (i_1_, …, i_m_) in the model, thus satisfying:

$$\begin{array}{c} X_{i_{1}i_{1k}}=X_{i_{2}i_{3k}}\cdot \cdot \cdot =X_{i_{n-1}i_{nk}}=1 \end{array}$$ (3)

That is the route which the tourist k passes. A shortest edge connecting point i_1_ or point i_m_ will be constructed in the model to meet the following requirements:

$$\begin{array}{c} X_{i_{1}j_{k}}=1 \end{array}$$ (4)

and

$$\begin{array}{c} W(\{i_{1},\cdot \cdot \cdot ,i_{m},j\})\leq W \end{array}$$ (5)

During the construction process, the shortest edge was searched. When searching for the shortest edge, a new route was selected until the farthest point was designated, thus forming a vehicle distribution route with a branch line.

## Research process

### Optimization analysis of tourism routes model flow chart

**Fig 2:**
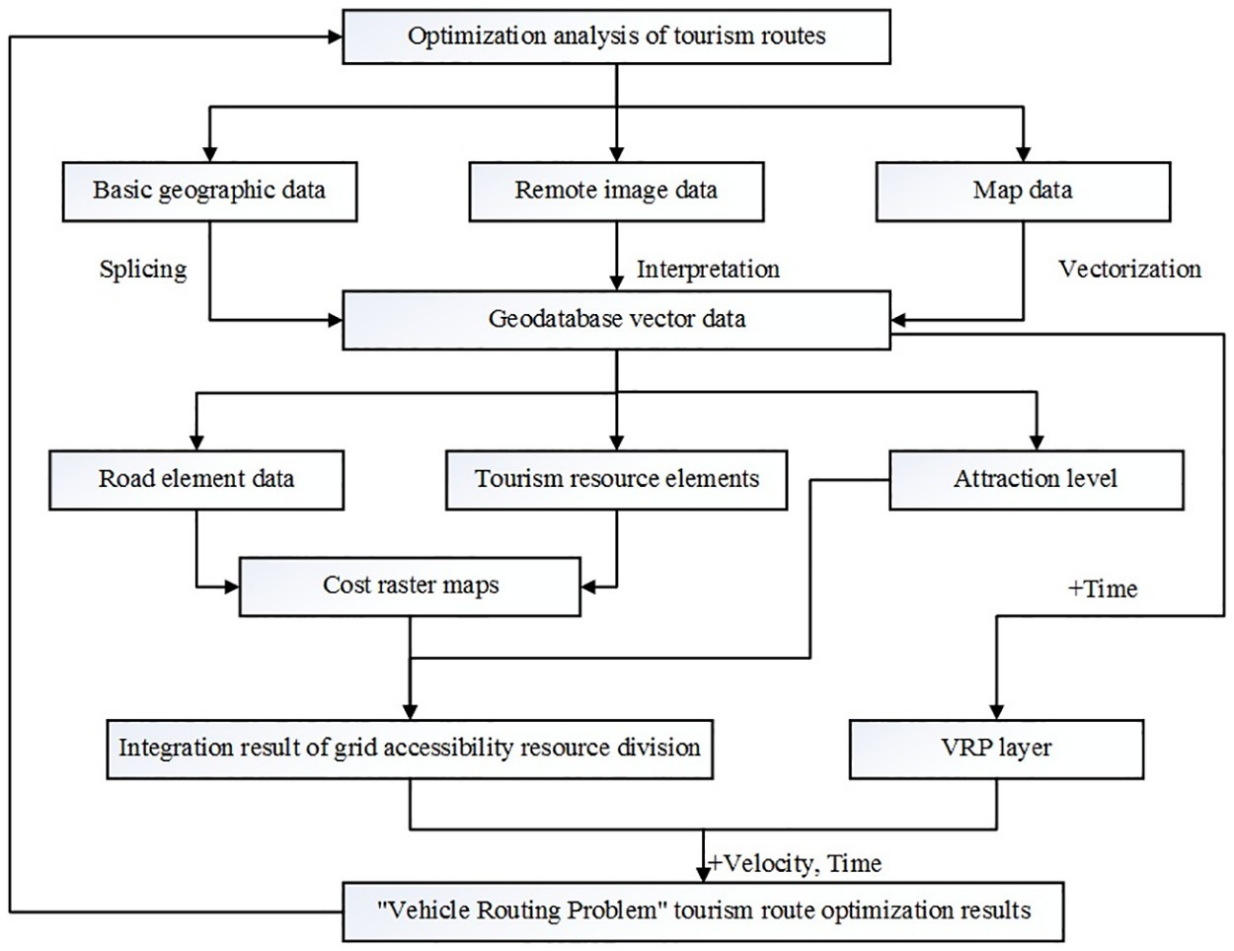
Optimization analysis of tourism routes model flow chart.

To achieve the optimization of tourism routes, it is necessary to conduct an analysis of the integration of tourism resources, achieve the maximum development of these resources through integration, strive for more favorable opportunities, and then optimize the routes for different themes and different days according to the results of the integration. First, a vector geodatabase was constructed in ArcGIS after the processing of basic geographic data splicing, remote sensing image data interpretation, and map data vectorization. The database contained road element data, node data, street dwelling point data, and tourism resource point elements. Then, for integrating the tourism resources, the road element data were subjected to classification and assignment, rasterization, raster and space overlay analysis; grid reclassification and weighting were combined with street resident data and tourism resource elements to generate a series of cost raster maps and conduct an accessibility evaluation. Finally, a time property was added to the constructed database, a VRP layer was created, and the Driving Speed, Service Time, Earliest Start Time, Latest Start Time, and so on were set in the “Vehicle Delivery Model.” See Fig 2 for the optimization analysis of tourist routes model flow chart.

### Integration of tourism resources based on grid accessibility

Before optimizing the tourism routes, the geographical location of the tourist attractions and the traffic accessibility were used to integrate the tourism divisions. The tourism resources of different levels were divided into regions that uphold the tourism development characteristics of different regions, and fully express the tourism characteristics of each street and the relationship between regions. This served to quantify the degree of connection between scenic spots, regions, the attractions, and the area. The purpose was to formulate reasonable tourism routes for tourists, enhance regional tourism development strategies, and provide strategies for the coordinated development of the tourism industry.

For the 217 tourist attractions in Lushunkou District, the “Cost Allocation” tool was used in the Spatial Analyst Tools module of ArcGIS to perform service zoning; the results indicated that 181 scenic spots have their own service areas, and the area of each service area is different. The more concentrated the scenic spot (such as Dengfeng Street, Market Street, and Desheng Street), the smaller its service area, while the more sparse the scenic spot (such as Beihai Street and Shuangdaowan Street), the larger the service area.

Scenic spots with a high comprehensive evaluation level of tourism resources have advantages in terms of popularity, attractiveness, and service level; therefore, when integrating tourism resources, such resources were used in this study to integrate scenic areas with a low evaluation score. Through a comprehensive analysis of the distribution of tourism resources in Lushunkou District, this study used six time thresholds of 5 min, 10 min, 15 min, 20 min, 25 min, and 30 min to merge tourism resources layer by layer.

### Tourism route optimization process

To optimize tourism routes based on network analysis technology, we first built a basic GIS database. Existing tourist attractions were identified as stops, and a point passing from Dalian to Lushun was selected as the station. At these stops, tourists depart from the station to arrive at the stop for sightseeing, and finally return to the station. Using this approach, the “vehicle distribution model” was used to optimize the route and form an optimized plan for the tourism route.

The GIS database was built in the ArcGIS environment. The data included regional administrative district layers, regional traffic data layers, regional tourist attraction data. The VRP layers required for the network analysis were based on regional traffic data. The VRP layer required a time attribute. The traffic cost of the VRP network dataset was divided into three types: distance-based, speed-based, and time-based. Based on the distance cost, the spatial distance between the tourist attraction and the tourist attraction along the traffic road was mainly considered, while the road capacity was usually not considered. This was because, in practice, the travel mode considered was riding. Thus, the speed was set to 50 m/min and the travel time was calculated based on that information. After creating the VRP layer, we set the three required attributes of the station, the stop, and the route for analysis.

Regarding constant and data loading, in the “Vehicle Routing Problem,” the station indicated the departure point and the return point; this was designated the “base camp,” i.e., the place of return after a lap. In this study, the selection of the station was determined manually, because no existing identified station was available in the data considered for the present study. When carrying out the “Vehicle Routing Problem,” exit points of Lushun South Road, Lushun Middle Road, and Lushun North Road were selected as stations. The stop was a tourist attraction that tourists would pass through during the tour, which can be understood as “the place passing by.” The stops in this instance included different types of tourist attractions such as patriotic tours, popular science tours, sightseeing agricultural tours, natural scenery tours and other types of tourist attractions. When loading tourist attractions, the Service Time, or average sightseeing time of each attraction, was set to 120 min. The path was determined based on the travel time and order of spots in a tourism project. According to the opening and closing time of the attractions, we set the Earliest Start (departure) Time to 8:00 and the Latest Start (departure) Time to 10:00. Specifically, this window was used to designate the time at which the optimal number of attractions could be visited; otherwise the number of attractions visited on a certain day would be reduced. The Start Depot Service Time was set to 8:00 and the End Depot Service Time was set to 16:00; specifically, the tour time started at 8 o’clock and ended at 4 o’clock in the afternoon. Eventually, the one-day tour route and the two-day tour route were determined according to the time of the tour.

## Results and analysis

### Consolidated analysis of tourism resource service areas

The merger of tourism resource service areas generally prioritizes the regional traffic convenience of the integration of regional attractions; specifically, the process of the displacement of tourists between the beginning of the arrival and destination as well as the magnitude and extent of the displacement are related to the traffic road network. A highly developed transportation network can promote the integration of resources; if transportation is inconvenient, tourists will seek other destinations. Only scenic spots with convenient transportation can enhance competitiveness and occupy a favorable source for tourists. More specifically, only scenic spots with convenient transportation can enhance competitiveness and attract more tourists. In summary, the service areas of tourist attractions are integrated according to the convenience of transportation. When the time distance between adjacent tourist attractions is less than the time threshold, the two tourist attractions are integrated, and the time threshold gradually increases; this aids in achieving the gradual integration of the tourism area until the integration of tourism resources.

**Fig 3:**
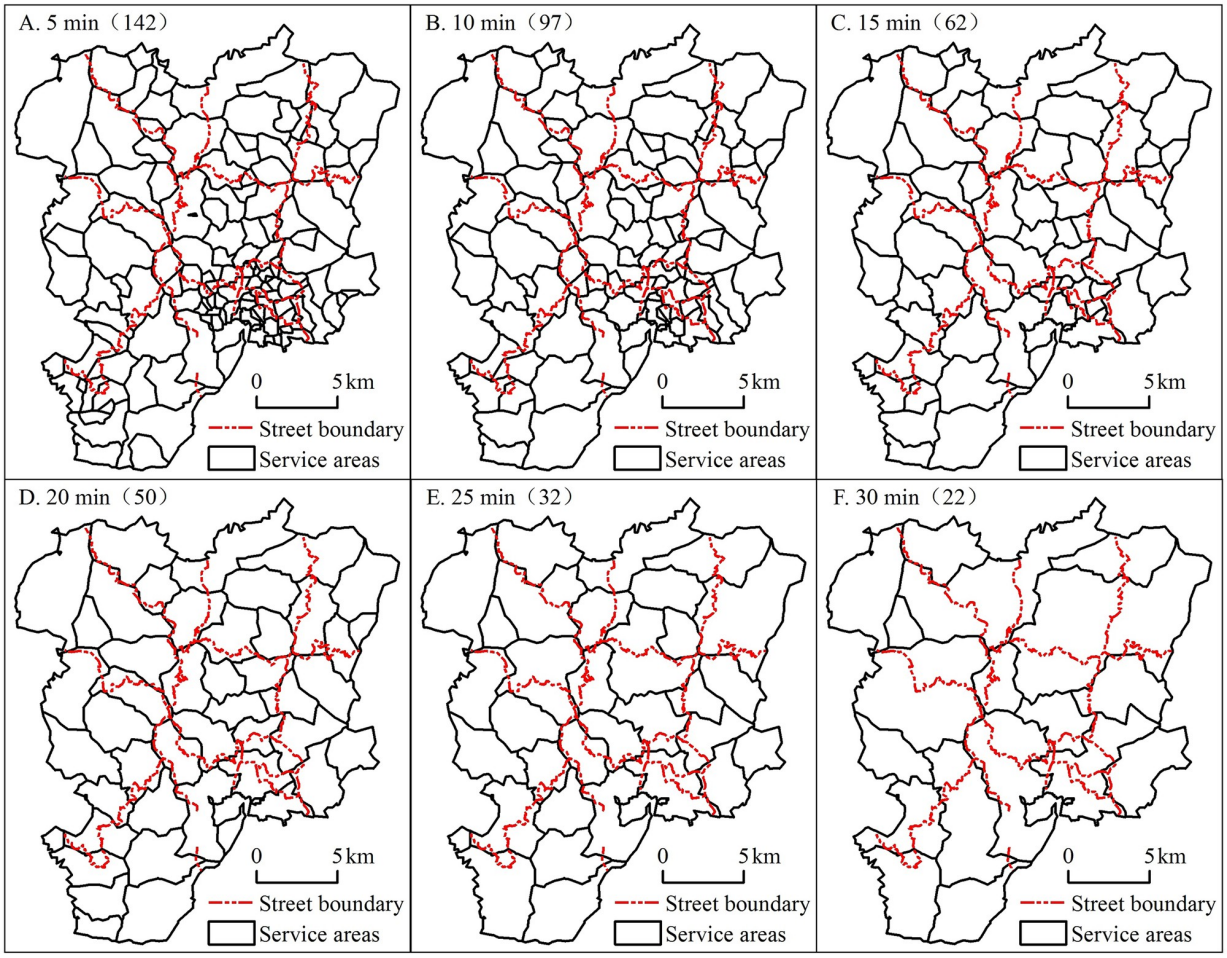
Tourist attractions service range integration evolution.

In general, the larger the time interval, the greater the degree of integration, and the smaller the number of service areas. The more concentrated the attractions, the smaller the distance, and the faster the integration speed. The distances between highly concentrated scenic areas such as Dengfeng Street, Market Street, and Desheng Street are relatively small, and the road network density is large; hence, their merging speed is high. For low concentration areas, the merging speed is low, such as in the scenic locations of Beihai Street and Sanjianbao Street Fig 2 At the threshold time of 5 min, the number of service areas decreased from 181 to 142; at 10 min, the number decreased to 97; at 15 min, the number decreased from 97 to 62; at 20 min, the number decreased to 50, and at this time, scenic spots with a high density of tourist attractions were successively integrated with those with a low density of such attractions. By 25 min, the number decreased to 32, mostly leaving only the key scenic areas; at 30 min, the number further decreased to 22, and at this time, the majority of the scenic spots were integrated, but the pattern did not change substantially, as only the service areas with smaller surrounding areas were integrated. See Fig 3 for tourist attractions service range integration evolution.

For further discussion and research, it is imminent to choose a suitable service area range, as different time thresholds integrate different numbers of service areas, and the service area range may be excessively large or excessively small to integrate tourism resources. The time thresholds of 5 min, 10 min, 15 min, and 20 min were too short, as the service area was divided into excessively small zones. The number of service areas was greater than or close to 50, and the distance between scenic areas was small; thus, the service area was dense, which did not reflect the proper spatial distribution of the scenic locations. In contrast, when the time threshold was 30 min, the number of service areas was small and many important attractions were integrated. However, the service area itself was sparse, and therefore the characteristics between attractions were not adequately reflected to a certain extent. When the time threshold was 25 min, the number of service areas was 32, the range was moderate, and analysis could be easily performed. Hence, for further analysis, this study selected 25 min as the time threshold to divide the service area into the basic unit of tourism resource integration.

### Tourism resource integration

**Fig 4:**
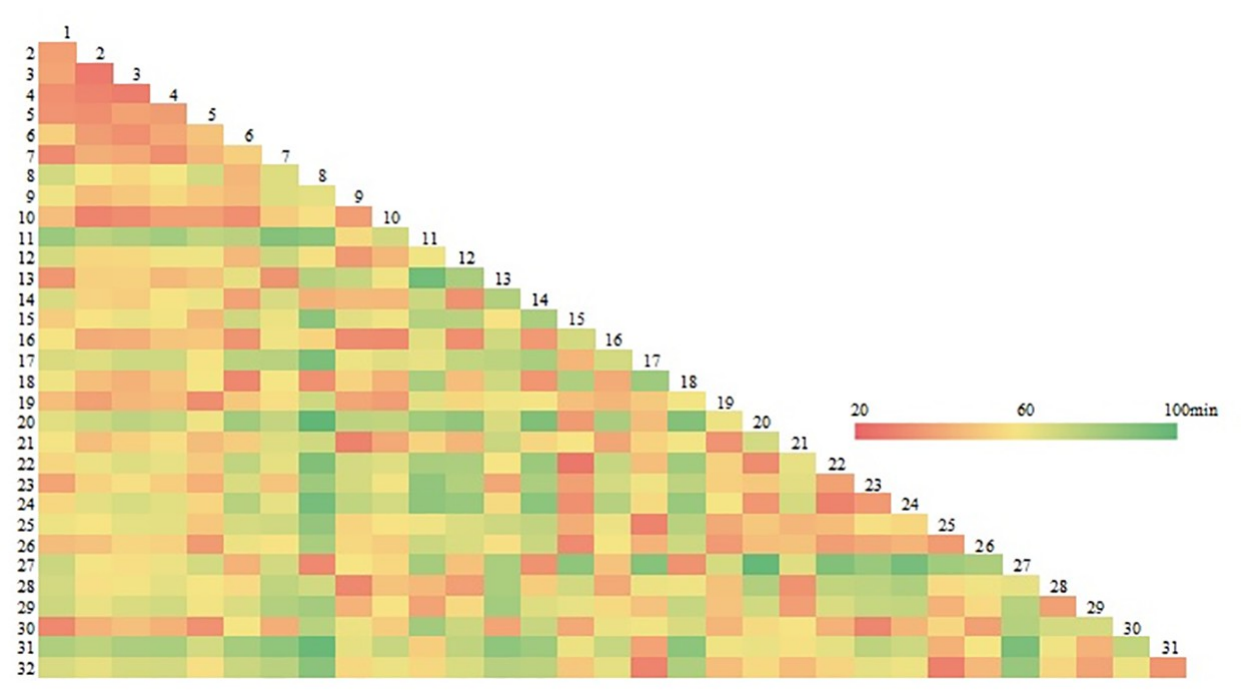
Matrix table of travel time between attractions after the integration of tourism resources.

Different levels of tourist attractions were designated, as different levels and categories of attractions had different functions, features, and service scopes. The attractions with lower ratings were integrated by the attractions with higher ratings. The scenic locations in Lushunkou District were integrated into the historical and cultural core area (Taiyanggou Scenic Area, East Jiguan Mountain Scenic Area, and Navy Camp Meeting Place), museum garden scenic area, natural scenic locations (Laotieshan Natural Scenic Area and Baiyinshan Scenic Area), military cultural area (Baiyushan Natural Scenic Area and 203 Scenic Area), agricultural sightseeing area (Laotieshan Agricultural Sightseeing Area, Beihai-Shuangdaowan Street Agricultural Sightseeing Area, and Sanjianbao-Great Wall-Longtou Agricultural Sightseeing Area), and human landscape area (Yantai-Dalian Railway Ferry Port Tourist Area). There were 6 types of scenic areas, including 12 scenic areas. The travel distance of the 32 service areas in these 12 scenic areas is shown in Fig 4; this provides a basis for route optimization through integrated analysis.

### Optimization results of tourism routes

Based on the “Vehicle Routing Problem” to optimize the tourism route as well as the popular science tours, patriotic tours, leisure tours, agricultural sightseeing tours, and the length of travel time, the travel routes for one-day tours and two-day tours were designed. The optimized sightseeing sequence and time are shown in Table 3. Through the integration of tourism resources, the tourism revenue was maximized.

**Table 3:** Attractions and tour schedule.

Route type	Attraction name	Arrival time	Departure time
**Popular Science One-Day Tours**	Snake Island Old Iron Mountain Nature Museum	8:00:00	10:00:00
	Gramophone Museum of Dalian Gule Building	10:22:14	12:22:14
	Lushun Museum Branch	12:28:27	14:28:27
	Museum of the Former Site of the Kwantung Army Headquarters	14:36:49	16:36:49
**Popular Science Two-Day Tours**	Snake Island Old Iron Mountain Nature Museum	8:00:00	10:00:00
	Lushun Museum Branch	10:09:43	12:09:43
	Lushun Museum Scenic Area	12:12:16	14:12:16
	Coin Museum	14:13:37	16:13:37
	Museum of the Former Site of the Kwantung Army Headquarters	8:00:00	10:00:00
	Lushun Snake Museum	10:09:49	12:09:49
	Lushun Museum Branch	12:20:02	14:20:02
	Gramophone Museum of Dalian Gule Building	14:26:15	16:26:15
**Patriotic One-Day Tours**	South Bullet Depot large artillery position	8:00:00	10:00:00
	Lushun Wanzhong Tomb Memorial Hal	11:07:25	13:07:25
	Lushun Japan-Russian Prison Site Museum	13:45:35	15:45:35
	East Jiguan Mountain Scenic Area	16:46:03	18:46:03
**Patriotic Two-Day Tours**	Friendship Tower	8:00:00	10:00:00
	Sun Valley Scenic Area	11:08:52	13:08:52
	Dalian brave the journey to Northeast Folk Culture Village	13:23:25	15:23:25
	Lushun Soviet Army Memorial Hall	15:47:53	17:47:53
	Lushun Wanzhong Tomb Memorial Hall	8:00:00	10:00:00
	Lushun Japan-Russian Prison Site Museum	10:38:10	12:38:10
	East Jiguan Mountain Scenic Area	13:38:38	15:38:38
	South Bullet Depot large artillery position	17:48:57	19:48:57
**Leisure One-Day Tours**	Laotie Mountain Nature Reserve	8:00:00	10:00:00
	Lushun National Forest Park	13:30:06	15:30:06
	Baiyu Mountain Scenic Area	17:39:22	19:39:22
**Leisure Two-Day Tours**	Baiyu Mountain Scenic Area	8:00:00	10:00:00
	Lushun National Forest Park	12:09:16	14:09:16
	203 Scenic Spots	14:41:46	16:41:46
	Laotie Mountain Nature Reserve	8:00:00	10:00:00
	Huanghai Bohai Demarcation Line Scenic Spotl	14:48:07	16:48:07
**Agriculture Sightseeing One-Day Tours**	Lushun Tianmu Hot Spring	8:00:00	10:00:00
	Lvxin Ecological Park	13:26:04	15:26:04
	Niu Niu Jiu Jiu Strawberry Picking	16:00:49	18:00:49
**Agriculture Sightseeing Two-Day Tours**	Lushun Tianmu Hot Spring	8:00:00	10:00:00
	Tide Mouth Bath	10:58:47	12:58:47
	Lvxin Ecological Park	15:26:04	16:41:46
	Laotie Mountain Nature Reserve	8:00:00	10:00:00
	Huanghai Bohai Demarcation Line Scenic Spotl	14:48:07	16:48:07

The popular science tour is mainly a museum tour, making full use of the popular science education function and mystery of the museum to attract more tourists. The Lushun Museum in the popular science tour route was built by the Japanese colonial authorities in 1917. The Snake Museum is the largest exhibition hall of snakes and amphibians and reptiles in Asia, the Gramophone Museum of the Gule Building is the most vivid business card of the 100-year-old Lushun, and it is also the only museum in mainland China that focuses on phonographs. The Former site of the Kwantung Army Headquarters is the Army Artillery Department of the Russian Army Kwantung Prefecture. These museums and building sites have strong educational significance. Relying on museums and celebrities’ former residence, the museums and building sites are integrated and developed to promote the development of cultural relic exhibitions, art creation, and tourist souvenir trading. To render the region even more tourist-friendly, a cultural gathering center should be built for academic seminars, art exchanges, and recreation.

Patriotic tours mainly include historical and cultural attractions. Among them, the East Jiguan Mountain Scenic Area is a national 4A-level scenic spot, i.e., a provincial-level cultural relic protection unit, and is known as an “open-air museum.” The patriotism education characteristic of these scenic spots should be fully utilized to attract more tourists. Notably, in the design of the patriotic tour route, because the distance between the attractions is considerably large and the travel speed is set to 50 m/min, there is a long commute from one attraction to another, resulting in the tour time exceeding 16:00. In the actual tour, however, the time from one scenic spot to the next will be shortened; hence, the error regarding the tour time can be ignored.

Leisure tours mainly constitute natural attractions. With its cherished birds and the natural sea boundary, Lushunkou District is rich in such attractions. These attractions should be fully utilized to attract more tourists. A route covering such attractions would include the Laotie Mountain Nature Reserve, the boundary between the Yellow Sea and the Bohai Sea, and the Laotieshan Lighthouse. These locations are highly enriched in natural resources. An integrated development of these natural tourism resources can enhance the domestic and foreign influence of the natural scenic spots in Lushunkou District. A combination of bird watching, sightseeing, and holiday entertainment, while enjoying the scenery, can provide an understanding of the key birds protected by the state, and thereby provide an enjoyable learning experience.

Sightseeing agricultural tours can include the Laotieshan Sightseeing Agricultural Area, Beihai-Shuangdaowan Street Sightseeing Agricultural Area, and Sanjianbao-Great Wall-Leading Street Sightseeing Agricultural Area. All these tourism resources all include cherry tourism, resorts, baths, hot springs baths, and other leisure tourism resources. The picking gardens and recreational villas with distinctive characteristics and unique charm are integrated to develop tourism resources. Farming, picking, and other leisure experience activities should be added, transformation of agricultural labor activities into artistic behaviors, the product cultural connotation and industrial added value should be improved, and a creative tourist agricultural area should be created that integrates the following: production and sale of creative agricultural products, folk experience, special cuisine, and wild entertainment.

## Conclusion

The tourism industry in Lushunkou District is developing rapidly, but there are obvious gaps compared with those around Dalian and other coastal cities; this is mainly because the spatial distribution of tourism resources is scattered, the overall image is not prominent, the tourism route is singular, and the business philosophy is one-sided. To achieve the sustainable development of the tourism industry, this study uses 217 tourism monomers in Lushunkou District and deploys the accessibility evaluation model and Vehicle Routing Problem to divide the functions of tourism resources, reasonably integrate the tourism resources, and build a perfect tourism route that realizes the maximization of tourism economic value and the comprehensive benefits to be realized as a consequence of tourism-based such development of Lushunkou District. Our specific conclusions are as follows:

1. The integration analysis of tourism resources through the accessibility model shows that tourism resources are integrated into a historical and cultural core area composed of the Taiyanggou Scenic Area, East Jiguan Mountain Scenic Area, Navy Camp Meeting Place and Dengfeng Street, museum garden scenic spot composed of Lushun Museum Garden Scenic Spot, natural scenic spot composed of Laotieshan Natural Scenic Spot and Baiyinshan Scenic Spot, military cultural zone composed of Baiyushan Natural Scenic Spot and 203 scenic spot, old Tieshan Mountain Sightseeing agricultural area, Beihai-Shuangdaowan Street Sightseeing Agricultural Area, and Sanjianbao-Great Wall-Leading Street Sightseeing Agricultural Area, which is a cultural landscape area composed of the Yantai-Dalian Railway Ferry Port Tourist Area.
2. To optimize the tourism routes, following activities should be created based on the “Vehicle Routing Problem”: one-day and two-day popular science tours, one-day and two-day patriotic tours, one-day and two-day leisure tours, and one-day and two-day agricultural sightseeing tours. Through the integration of tourism resources and route optimization, the purpose of tourism resources integration and development can be realized, and a new method and mode of tourism resources integration are constructed, which provide a basis and reference for the integration of tourism resources in similar cities.
3. This research provides an overall idea framework and technical method for tourism route optimization and has been verified using Lushunkou District as the case study. The technical system can be replicated and promoted, and it can serve as an important reference for the government to develop the tourism industry, and for travel agencies and tourists to design tourism routes. In this study, primarily land areas were considered; some sea area such as Pig Island, Huping Island, Snake Island, and Sea Cat Island were not involved in the integration of tourism resources and route optimization. This was owing to the problems encountered during the research process and the derivation of solutions, research time involved and data occupancy. Therefore, in future research, it is imperative to integrate tourism resources in the sea area for the overall integration of tourism resources to achieve the goal of “overall land and sea development.”

## Supporting information

S1 File (RAR)

## References

[1] World travel and tourism council. Available online: https://www.wttc.org/economic-impact/. accessed on 27 April 2021.

[2] Han Y, Guan H, Duan J. Tour route multiobjective optimization design based on the tourist satisfaction. Discrete Dynamics in Nature and Society.2014, 2014. doi: 10.1155/2014/603494

[3] Magwire Păcurar C.M.; Albu R.-G.; Păcurar V.D. Tourist route optimization in the context of Covid-19 pandemic. Sustainability. 2021, 13, 5492.

[4] Tsai M-C. Developing a sustainability strategy for Taiwan’s tourism industry after the COVID-19 pandemic. PLoS ONE. 16(3): e0248319. doi: 10.1371/journal.pone.0248319 33705479 PMC7951935

[5] Lau G. Understanding tourist movement patterns in a destination: A GIS approach. Tourism and Hospitality Research. 2006, 7, 39–49. doi: 10.1057/palgrave.thr.6050027

[6] Xiongbin W, Hongzhi G, Yan H, et al. A tour route planning model for tourism experience utility maximization. Advances in Mechanical Engineering. 2017, 9(10).

[7] Wenrui Z. Application of an improved ant colony algorithm in coastal tourism route optimization. Journal of Coastal Research. 2019, 98(sp1): 84–87. doi: 10.2112/SI98-021.1

[8] Jincao W, Lijun F, Yawen Y. Multi-objective optimization model based on travel directions. Mathematics in Practice and Theory. 2016, 46(15): 97–104.

[9] Liang S, Jiao T, Du W, Qu S. An improved ant colony optimization algorithm based on context for tourism route planning. PLoS ONE. 16(9): e0257317. doi: 10.1371/journal.pone.0257317 34529729 PMC8445481

[10] Pan Q, Wang X. ndependent travel recommendation algorithm based on analytical hierarchy process and simulated annealing for professional tourist. Applied Intelligence. 2018, 48, 1565–1581. doi: 10.1007/s10489-017-1014-0

[11] Krumm J, Gruen R, Delling D. From destination prediction to route prediction. Journal of Location Based Services. 2013, 7, 98–120. doi: 10.1080/17489725.2013.788228

[12] Zheng W, Huang W, Li Y. Understanding the tourist mobility using GPS: Where is the next place? Tourism Managemant. 2017, 59, 267–280.

[13] Wei S, Yanfeng S, Jingyi S, Zhiwei Y. Modeling and research on tourism route planning. Mathematics In Practice And Theory. 2016, 46(15): 115–124.

[14] Wolfgang W, Hefele A, Herzog D. Recommending a sequence of interesting places for tourist trips. Information Technology and Tourism. 2017, 17, 31–54. doi: 10.1007/s40558-017-0076-5

[15] Gill N, Bharath B. Identification of optimum path for tourist places using GIS based network analysis: A case study of New Delhi. Journal of Sports and Science. 2013, 1, 34–38.

[16] Ahmad S, Ullah I, Mehmood F, et al. A stochastic approach towards travel route optimization and recommendation based on users constraints using markov chain. IEEE Access. 2019, 7: 90760–90776. doi: 10.1109/ACCESS.2019.2926675

[17] Dantzig G.B.; Ramser J.H. The Truck Dispatching Problem. Management Science. 1959, 6, 80–91. doi: 10.1287/mnsc.6.1.80

[18] Songyi W, Fengming T, Yuhe S, Haolin W, Francesco A. Optimization of Vehicle Routing Problem with time windows for Cold Chain Logistics based on carbon tax. Sustainability. 2017, 9(5).

[19] Güneş Erdoğan. An open source spreadsheetsolver for Vehicle Routing Problems. Computers and Operations Research. 2017,84.

[20] Lusheng P. Personalized recommendation of tourist information mining of Gansu attractions tourism. Journal of Lanzhou University of Arts and Science (Natural Sciences). 2018, 32(06): 82–87.

[21] Yu N. Research on spatial structure of tourism industry of northwestern region under regional cooperation perspective. Lanzhou University. 2013.

[22] Yuezheng L, Fuhai Z. Design and development about regional tourist net of Benxi city. Economic Geography. 2002(04): 497–500+505.

[23] Le Y, Yongming W. A GIS-based study on tourism resource functional divisions and routes planning in Chuzhou city. Geomatics and Spatial Information Technology. 2010, 33(01): 31–33+41.

[24] Abubakar E, Idoko O, Ocholi O. Efficient tour planning for tourist sites visitation in Lokoja, Nigeria: A multi-scenario analysis using GIS. Journal of Geographic Information System. 2017, 9, 59–81. doi: 10.4236/jgis.2017.91005

[25] Guisong M. The preliminary thinking of the design about geologic tour routes at Shongshan mountain. Journal of Henan Education Institute (Natural Science). 2001(04): 37–39.

[26] Shilin Z, Jian R, Bo L, Xianchun G. The application of the shortest path algorithm in the tourist route planning-with Mt. LuShan as an example. Science of Surveying and Mapping. 2008(05): 190–192.

[27] Xiao A, Minhua G, Xiaolin M. Design and development of Guangyu tourism information system based on GIS. Anhui agricultural science bulletin. 2011,17(19): 145–149.

[28] Daiyin S, Jingjing L, Xin Y, Xuan L, Yiping S, Yuerong D. Optimal design of tourist routes in Shenyang World Expo Garden Scenic Spots based on intelligent algorithms. Application of Mechanics-electronics Technology. 2019, 50(01): 176.

[29] Zhuo X, Chen K, Chen Y, Zhou B. Using RMP analysis in Huludao City creative park planning. Planners. 2015, 31(03): 40–45.

[30] Jinghu P, Yibo C. Tourism regionalization in China based on spatial accessibility of A-grade scenic spots. Scientia Geographica Sinica. 2014, 34(10): 1161–1168.

[31] Lin L. Research and design on GIS-based logistics distribution system. Huaqiao University. 2006.

